# Effects of Head-Neck Position and Ground Surface on Gait Kinematics in Ridden Brazilian Criollo Horses

**DOI:** 10.3390/ani16071043

**Published:** 2026-03-29

**Authors:** Natália Almeida Martins, Laura Patterson Rosa, Maria Inês Frank, Camila Feil Dellbrigge, Weliton Luiz Marafon, Letícia Bisso Paz, Caio Henrique Schmidt, Flávio Desessards De La Côrte

**Affiliations:** 1 Departamento da Clínica e Cirurgia de Grandes Animais, Universidade Federal de Santa Maria, Santa Maria 97105-900, RS, Brazil; mariainesfrank@hotmail.com (M.I.F.); camilafeil6@gmail.com (C.F.D.); marafonweliton@gmail.com (W.L.M.); leticia.paz@acad.ufsm.br (L.B.P.); caiohenriqueschmidt@gmail.com (C.H.S.); delacorte2005@yahoo.com.br (F.D.D.L.C.); 2 Department of Veterinary Clinical Sciences, Lewyt College of Veterinary Medicine, Long Island University, Brookville, NY 11548, USA; laura.patterson@liu.edu

**Keywords:** gait analysis, kinematic, Criollo horse, head and neck position

## Abstract

The Criollo horse is prized for its endurance, yet its specific biomechanical signatures remain under-researched. This study investigated diagonal dissociation and support patterns in eleven clinically sound Criollo horses to understand the influence of head and neck position (HNP), surface compliance, and morphometry. Using high-speed video (120 fps) and kinematic analysis, we found that diagonal dissociation occurred in 85.24% of hoof contacts, while HNP1 showed a higher frequency of positive (hindlimb-first) dissociation and HNP2 had a higher incidence of negative (forelimb-first) dissociation (*p* = 0.0398). On soft ground, both HNPs predominantly exhibited hindlimb-first dissociation (*p* = 0.0446). Soft ground is also associated with the appearance of tripedal support, whereas hard ground presented more synchronous (non-dissociated) diagonal limb support. Thoracic monopedal and tripedal supports are weakly correlated with forelimb measurements, whereas pelvic monopedal and tripedal supports correlate with global body dimensions. All associations were weak to moderate (R^2^ ≤ 0.40). The prevalence of dissociated phases and the absence of a standard symmetrical trot suggest a distinct breed signature adapted for functional efficiency and stability required for its traditional working roles.

## Simple Summary

The Criollo horse is a symbol of South American tradition, valued for its endurance and comfortable ride. However, the science behind how these horses move is not yet fully understood. This study looked at eleven healthy Criollo horses’ gaits under different conditions, such as having a loose or flexed neck and moving on soft or hard ground. We found that these horses rarely move their diagonal legs in perfect synchrony. Instead, they usually land their pelvic limb slightly before their thoracic diagonal pair. This “split-second” difference, known as diagonal dissociation, is influenced by the ground surface and the horse’s head and neck position. Our findings suggest that the Criollo horse exhibits a gait pattern that may prioritize stability and energy saving over vertical suspension phases during locomotion. This could explain why they are known to be efficient for long workdays on farms and provide a smooth experience for the rider. Understanding these natural movement patterns helps breeders and veterinarians better support the health and performance of this breed.

## 1. Introduction

The Criollo horse is a South American breed developed from European stock horses that bred freely in South American nature for approximately 200 years. Today, the breed is highly valued in southern Brazil and its neighboring countries for its exceptional farm work ability, hardiness, and distinctive smooth trot. Beyond their significant economic role in cattle farming and cattle-working ability, Criollo horses are also prized for their locomotion efficiency and the comfort they provide to riders [1,2,3]. Despite these important functional attributes, quantitative analysis of the Criollo trot and respective environmental and individual factors (such as head and neck position, ground, and morphometric traits) influencing its biomechanics remains unexplored.

Locomotion efficiency is intrinsically linked to energy expenditure, which is greatly influenced by limb–ground contact patterns [4,5]. A crucial aspect is diagonal dissociation, defined as the temporal offset between the landing (touching the ground) or take-off (leaving the ground) of the diagonal limb pair or asynchrony in limb movements where the pelvic or thoracic limb touches the ground before its diagonal counterpart. This is a key biomechanical strategy that significantly reduces energy expenditure compared to synchronous ground contacts [6,7]. The diagonal limb pairing can be classified as synchronous, hindlimb-first (positive dissociation, often desirable in trotting breeds), or forelimb-first (negative dissociation) [8]. The degree of asynchrony in diagonal dissociation varies among individuals and gait types [8,9,10,11,12,13]. In sport horses, trot predominantly demonstrates negative dissociation, with variations depending on breed [8,13,14,15] and degree of trot collection [9]. Additionally, variations in diagonal dissociation are observed in horses performing advanced dressage movements [13], and conformational differences can also influence locomotion, balance and limb coordination [16,17], as observed in other species such as dogs [18]. This gap is especially relevant for the Criollo breed, in which conformation standards are well-established [2,19,20], yet its link to functional performance [21,22] is still understudied. External factors also play a role, as head and neck position (HNP) can alter diagonal dissociation [23] and surface properties can alter locomotion biomechanics. Hard surfaces can modify equine locomotor patterns by reducing stride frequency and increasing stride length to minimize vertical impact and deceleration through longitudinal compensation [24]. Despite this, there is a notable lack of research examining how surface conditions influence diagonal dissociation timing or patterns.

We evaluated the interaction between linear and temporal locomotor variables and environmental factors to better understand the breed’s unique locomotor patterns. Specifically, our study aimed to characterize diagonal dissociation during the ridden trot in the Brazilian Criollo horse. We further investigated the influence of two head and neck positions (HNPs) and ground hardness on dissociation patterns and evaluated the relationship of morphometric measurements and support sequences during the Criollo characteristic gait. We hypothesized that HNPs and ground hardness would significantly influence diagonal dissociation timing, while the morphometric measures of limbs would have important correlation with support times.

## 2. Materials and Methods

### 2.1. Animals

The animal study protocol was approved by the Institutional Ethics Committee of Universidade Federal de Santa Maria (CEUA-UFSM) (protocol code 6403240523, date of approval 25 September 2023). The study subjects were sourced from Santa Maria, Rio Grande do Sul, Brazil, a region characterized by flat terrain. Included horses belonged to the Criollo breed by parentage and were regularly ridden for farm and cattle work (average workload of two to three hours of work per day for each animal), having fundamental training as riding animals–yet no intermediate or advanced training in disciplines such as dressage or other competitions. These horses are managed in an extensive production system and primarily housed in open pastures. Typically, the animals begin a handling process (halter and saddle habituation) at approximately 18 months of age, with formal riding starting between two and three years. In their daily operational routine, the horses are employed for livestock handling, including cattle herding, inspections, and maintenance of fence lines.

To ensure the inclusion of clinically sound horses, excluding individuals exhibiting locomotion asymmetry consistent with lameness or musculoskeletal pathologies, an objective evaluation was performed using the Lameness Locator system (Equinosis, Columbia, MO, USA). Horses were considered sound and included in the study if their vector sum maximum did not exceed 8.5 mm, and both PMax and PMin values were less than 3.00 mm, following the manufacturer’s recommendations [25,26,27,28,29,30].

Eleven clinically sound individuals comprising 7 females (mean age = 7.7 y, S.D. ± 4.68) and 4 geldings/castrated males (mean age = 5.5 y, S.D. ± 1.73) were included in the study. All individuals were measured using a standardized equine stick for withers height (cm), and a weight tape (kg), with no differences observed between sexes (*p* > 0.05). Twenty morphometric traits were measured by a trained investigator (N.A.M.) for each horse in centimeters: height at withers, height at croup, height at the back, vertical thorax length (ribcage); chest and hindquarter width; and head, neck, back-to-loin, croup, horizontal body, scapula (shoulder blade), humerus (arm), radius (forearm), femur (thigh), tibia (gaskin) and respective forelimb/thoracic and hindlimb/pelvic cannon and pastern bone lengths using a measuring tape (Supplemental Table S1).

### 2.2. Experimental Data Collection

Video recordings were performed using a Xiaomi Mi A3 high-speed camera (Xiaomi Corporation, Beijing, China) at 120 frames per second (fps) and 1920 × 1080 resolution. The camera was mounted on a tripod set at a 1.2 m height and positioned 10 m away, parallel to the horse’s trajectory line, and oriented to capture the sagittal plane view. Horses were ridden by their usual trainers (a total of 6 riders, whose equipment was standardized as much as possible, using the typical Criollo saddle and standard bridle) in a straight line on either sand (soft ground; *N* = 5 horses) or compacted dirt (hard ground; *N* = 6 horses). The surface was considered soft when the hoof consistently sank into the sand substrate about 50% of the hoof wall height.

**Figure 1:**
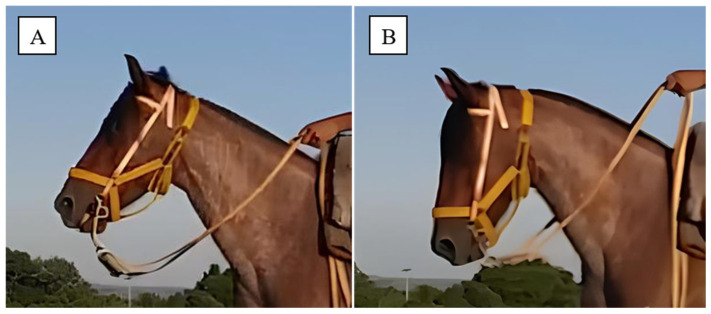
Example of the two evaluated head and neck positions (HPNs) adapted from [31], being (**A**) HNP1 = “Free” rein position (head and neck extended, with minimal rein contact from the rider) and (**B**) HNP2 = “Collected” position (head and neck elevated, with the poll as the highest point and the nose ~10° in front of the vertical line, achieved through light rein contact to encourage collection).

For each ground type, horses were ridden 5 times in each head and neck position (HNP), modified from [31], with at least two complete strides captured per pass, following established recommendations [32,33]. The HNPs were defined as HNP1 = “Free” rein position (head and neck at a relaxed position, with minimal rein contact from the rider) and HNP2 = “Collected” position (head and neck elevated, with the poll as the highest point and the nose ~10° in front of the vertical line, achieved through light rein contact to encourage collection) (Figure 1). To achieve the HNP2, riders applied light consistent tension on the reins (contact) combined with intermittent pressure from the heels against the horse’s ribcage. This simultaneous application of a forward-driving aid (legs) and a restraining aid (hands) prompted the horses to flex the poll and tuck the pelvis under the body mass.

### 2.3. Biomechanical Parameter Estimation

The times of contacts and take-offs for each hoof were obtained through a frame-by-frame video analysis using the Kinovea Version 2023.1.2 (https://www.kinovea.org). The percentages of hindlimb-first (positive), synchronous, and forelimb-first (negative) contacts were recorded for a total of 220 observations resulting from: 11 horses, ridden in two HPNs with 5 recorded videos per HPN, containing 2 strides per video, totaling 220 unique datapoints at 120 fps. Per horse, a total of 20 strides, and about 6400 individual frames were analyzed.

Diagonal dissociation was determined by calculating the time interval between one ground contact with the other in the diagonal pair or take-off of each diagonal pair using a custom formula within the spreadsheet. Diagonal dissociation time (Dt) in seconds was calculated as a function of the time when each hindlimb hoof first touches the ground (Ht) minus the time the forelimb hoof first touches the ground (Ft); or Dt = Ht − Ft [34]. To calculate take-off dissociation (also referred to as diagonal advanced lift-off), the formula was adapted to use the time of last ground contact instead of the first. Resulting data were recorded into a Microsoft Excel^®^ spreadsheet for organization and subsequent statistical analysis.

High-speed video recordings were also analyzed frame-by-frame to determine the sequence and temporal characteristics of limb support during locomotion. Each frame was classified into one of four support categories based on the number and configuration of limbs in contact with the ground: monopedal (single limb support, either thoracic or pelvic), bipedal (two-limb support, classified as lateral or diagonal), tripodal (three-limb support, categorized according to whether the thoracic or pelvic limb was in the swing phase), and quadrupedal (all four limbs in contact) [17]. For each recording, the duration of each support category was quantified, and the mean time spent in each support type was calculated to characterize the gait cycle (Supplemental Table S1).

### 2.4. Statistical Analysis

Statistical analysis was performed using JMP Student 18.0 (JMP Inc., Cary, NC, USA). Data were first evaluated for normal distribution through the Shapiro–Wilk test and subjected to logarithmic (log10) transformation when necessary. A Linear Mixed Model (LMM) was employed to assess factor effects on diagonal dissociation. The model included ground type (soft or hard), head and neck position (HNP1 or HNP2) and type of dissociation (landing or take-off) as fixed effects. To account for inherent variability and avoid individual bias due to repeated measures, individual horse and rider were included as random effects. The model was fit by Maximum Likelihood (ML). The significance threshold (alpha) for all analyses was set at *p* ≤ 0.05.

We analyzed the relationships between support types, morphometric measurements and HNP/ground type measurements using the coefficient of determination (*R*^2^), and the significance determined via Analysis of Variance (ANOVA) for normally distributed data and Wilcoxon-Kruskal–Wallis for non-normal data. For significant associations (*p* ≤ 0.05), a post hoc analysis using Tukey’s HSD or Steele–Dwass was performed to derive parameter variation.

## 3. Results

**Figure 2:**
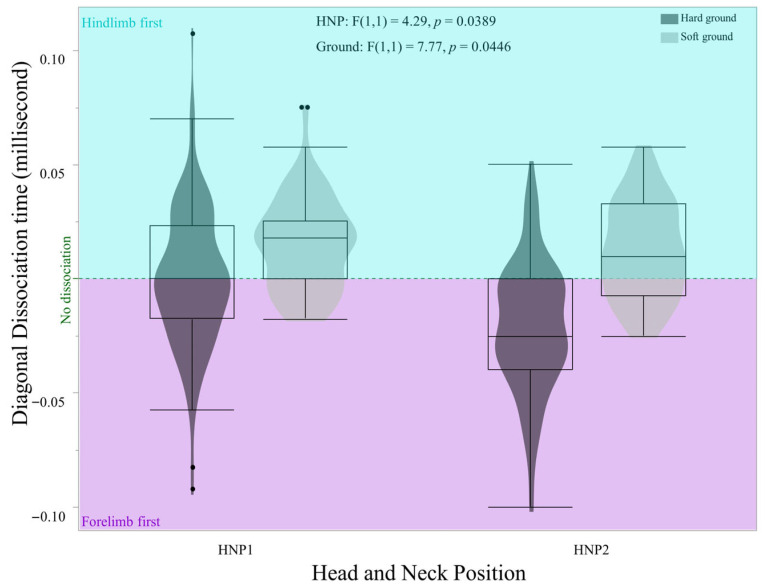
Distribution of diagonal dissociation (in milliseconds) by HNP and ground hardness for HNP1 = free rein, HNP2 = Collected position, hard ground (dark gray) and soft ground (light gray) for 11 Criollo horses. The areas represent positive dissociation (hindlimb-first, light blue) and negative dissociation (forelimb-first, purple), with the 0 value being “no dissociation” (green dotted line).

The head and neck position (HNP) significantly influenced diagonal dissociation during the trot in this cohort of Criollo horses. HNP1 demonstrated a higher incidence of positive (hindlimb-first) dissociation, while HNP2 had a predominance of negative (forelimb-first) dissociation (F (1, 1) = 4.29, *p* = 0.0398). Specifically, on soft ground, both HNPs exhibited a predominant hindlimb-first dissociation (F (1, 1) = 7.77, *p* = 0.0446). On soft ground, positive dissociation was observed in 90% (HNP1) and 86% (HNP2) of contacts, whereas on hard ground, negative dissociation prevailed with 43.33% (HNP1) and 68.33% (HNP2) of contacts. Synchronous contacts (no-dissociation) on hard ground occurred in 22.5% (HNP1) and 19.16% (HNP2) of the observed contacts, a considerable increase from 15% (HNP1) and 11% (HNP2) on soft ground (Figure 2).

**Figure 3:**
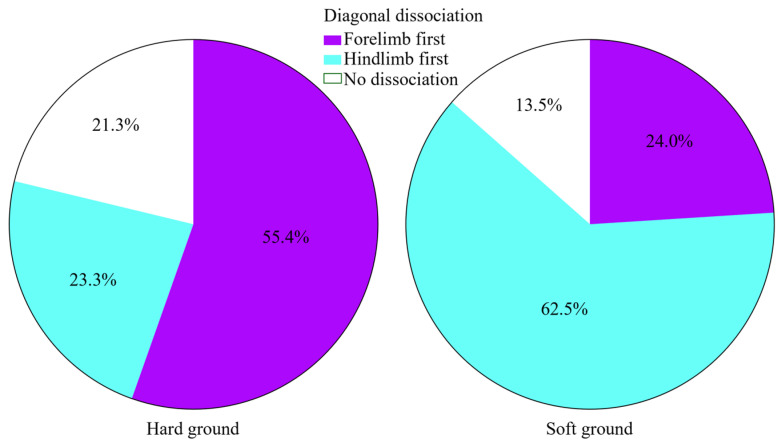
Distribution of ground type (hard versus soft) and respective relative diagonal dissociation observations (in percentage of the total diagonal dissociations). While the hindlimb-first occurs more frequently in soft ground, the hard ground had a higher percentage of forelimb-first and no-dissociation moments.

Not accounting for HNPs, hindlimb-first dissociation was notably higher on soft ground (62.5%), and no dissociation was less observed (13.5%). Hard ground conditions led to a higher percentage of forelimb-first contacts and an increase in synchronous/no-dissociation contacts (Figure 3).

Ground surface significantly influenced pelvic monopedal support (χ^2^ (1) = 8.89, *p* = 0.0029), where horses exhibited a 5.71% (95% CI: 2.61% to 11.39%) higher percentage of pelvic monopedal support when moving on soft compared to hard ground (Z = −2.94, *p* = 0.0032). In contrast, thoracic triple support had a higher (χ^2^ (1) = 4.35, *p* = 0.0369) incidence on hard ground compared to soft ground (Z = 2.04, *p* = 0.041). We observed significant rider effects for thoracic triple support (χ^2^ (6) = 13.65, *p* = 0.0369). However, post hoc pairwise comparisons did not identify differences between individual riders (*p* > 0.05), likely due to the limited sample size per rider.

**Figure 4:**
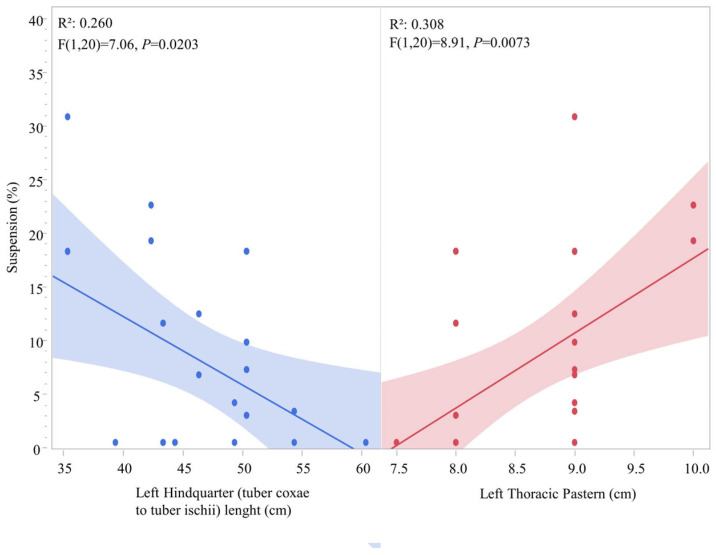
Correlation of suspension percentage per observation (horse/HNP) relative to pelvic length (tuber coxae to tuber ischii) and left thoracic pastern morphometric variables, where each point represents a single datapoint of an individual horse ridden in either HNP1 or HNP2. Measurements are in centimeters (cm).

Morphometric predictors also demonstrated significant correlations with type of support variables. A weak negative relationship was observed between pelvic length and suspension, where smaller pelvic length was correlated with increased suspension phases in this cohort (*R*^2^ = 0.260; F (1, 20) = 7.06, *p* = 0.0203). Suspension also demonstrated a weak-to-moderate positive correlation with the thoracic pastern, where an increase in this measurement was associated with an increase in the suspension variable (*R*^2^ = 0.308; F (1, 20) = 8.91, *p* = 0.0073) (Figure 4).

**Figure 5:**
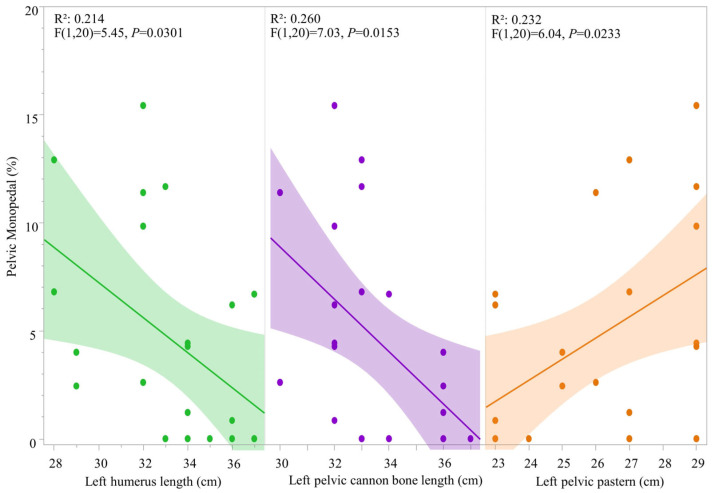
Correlation of pelvic monopedal percentage per observation (horse in both HNPs) relative to humerus length, pelvic cannon bone and pelvic pastern morphometric variations, where each point represents a single datapoint of an individual horse ridden in either HNP1 or HNP2. Measurements are in centimeters (cm).

Pelvic monopedal support also showed a significant weak negative correlation with the humerus/arm length (*R*^2^ = 0.214; F (1, 20) = 5.45, *p* = 0.0301) and pelvic cannon bone length (*R*^2^ = 0.260; F (1, 20) = 7.03, *p* = 0.0153) and a weak positive correlation to pelvic pastern length (*R*^2^ = 0.232; F (1, 20) = 6.04, *p* = 0.0233) (Figure 5).

**Figure 6:**
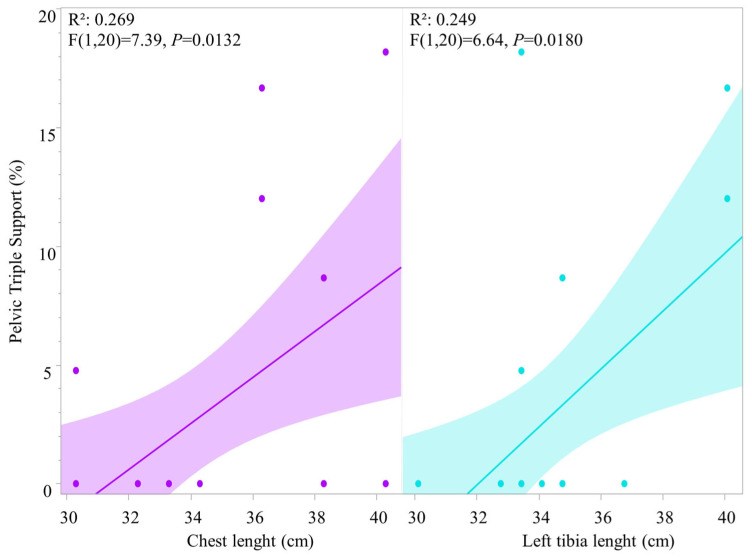
Correlation of pelvic triple support percentage per observation (horse in both HNPs) relative to chest and tibia length morphometric variations, where each point represents a single datapoint of an individual horse ridden in either HNP1 or HNP2. Measurements are in centimeters (cm).

Pelvic triple support has a weak, yet significant positive correlation with both chest length (*R*^2^ = 0.269; F (1, 20) = 7.39, *p* = 0.0132) and tibia length (*R*^2^ = 0.249; F (1, 20) = 6.64, *p* = 0.0180) (Figure 6).

**Figure 7:**
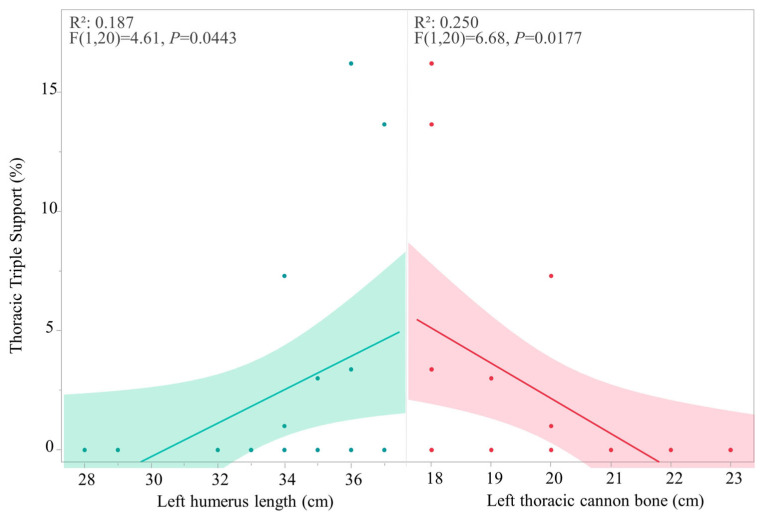
Correlation of thoracic triple support percentage per observation (horse in both HNPs) relative to humerus length (arm) and thoracic cannon bone morphometric variation, where each point represents a single datapoint of an individual horse ridden in either HNP1 or HNP2. Measurements are in centimeters (cm).

Thoracic triple support demonstrated a weak association with arm length (*R*^2^ = 0.187, F = 4.60, *p* = 0.0443) and thoracic cannon bone (*R*^2^ = 0.250, F = 6.67, *p* = 0.0177) (Figure 7).

## 4. Discussion

We demonstrate that Brazilian Criollo horses, similar to other breeds, may present diagonal dissociation as a strategy to minimize impact and optimize energy expenditure during ground contact in the trot [6,35]. While the definition of trot is a symmetrical two-beat locomotion pattern with diagonal ground contacts intercalated by suspension phases, a model largely derived from Thoroughbred and Warmblood studies, our data demonstrate that the Brazilian Criollo horse operates under a distinct biomechanical pattern. The consistent diagonal dissociation observed suggests that gait symmetry in this working breed may be more plastic than previously theorized. The overall synchronous contact occurrence was 14.76% of the total diagonal strides, comparatively lower than in other trotting breeds [36], especially relative to the high incidence of hindlimb-first dissociation, which may contribute to the riding comfort experienced in the trot of the Criollo breed. This divergence indicates that the Criollo has likely developed a specialized locomotor signature, possibly optimized for stability and endurance as these are desirable, selected traits by breeders [1,2,3]. Fully synchronous hoof–ground contacts are generally considered undesirable due to increased energy loss compared to hindlimb-first dissociations [6,7] and greater impact transmission to the rider, which is a key criterion for evaluating riding comfort in Brazilian breeds. However, in arena settings, some show horse breeds are selectively trained for a predominantly synchronous diagonal timing to emphasize a “pure” trot appearance, contrasting treadmill or field conditions where natural hindlimb-first dissociation prevails [6,36].

Results suggest that HNP and ground type influence diagonal dissociation patterns and timing. HNP1 results in more synchronous contacts on soft ground, while HNP2 exhibits more synchronous contacts on hard ground. Reduced impact forces experienced on soft ground might allow the horse in HNP1 to perform a less collected trot [9]. Furthermore, the increased collection in HNP2 on hard ground might be a compensatory mechanism to manage impact, potentially leading to a higher proportion of synchronous contacts. While hindlimb-first dissociation is biomechanically advantageous by reducing mechanical energy losses during hoof–ground collisions and improving trunk stability [6,7], its pattern can be altered by external factors. It is important to acknowledge that Criollo horses, predominantly used for farm work, differ from classical dressage breeds that are specifically trained for forehand elevation and increased collection, leading to improved self-carriage [37]. While self-carriage—the horse’s ability to move with balance and efficiency without constant rider intervention—is vital for optimal energy efficiency in any discipline [38], the HNP2 collection observed in Criollo horses is adapted to their working demands rather than classical dressage principles.

HNP2 is generally considered beneficial for working horses as it preserves anatomical flexibility, enhances locomotion, and potentially reduces injury risk, often by minimizing synchronous contacts [9]. The presence of diagonal dissociation creates a phase lag between the longitudinal components of the ground reaction forces from the fore and hindlimbs within a diagonal pair. This phase lag is crucial because it contributes to a reduction in the cumulative magnitude of impact peaks, as well as decreased breaking and propulsive peak forces [6,7]. Consequently, longitudinal accelerations of the body are mitigated, leading to a more even distribution of impact forces across bones and joints, which could potentially reduce the incidence of long-term work-related lesions in Criollo horses. Our results corroborate previous reports by showing fewer synchronous contacts in HNP2 compared to HNP1. Soft ground seems to also promote positive (hindlimb-first) dissociation. Surface properties significantly modulate equine kinematics and injury risk, e.g., hard surface elevates ground reaction forces (GRF) leading to increased subchondral bone impact, joint hyperextension, and chronic orthopedic wear [39,40,41,42]. The collected HNP2 might have a greater influence from the ground on dissociation patterns than HNP1, possibly due to the horse’s altered biomechanics under collection [9]. While the effects of head and neck positions (HNPs) on gait kinematics are well-documented in dressage and sport horses [8,9,10,11,12,13], our study fills a critical gap by providing the first quantitative kinematic evaluation of the Criollo trot under varying HNPs and ground conditions. Yet, it is difficult to untangle the effect of ground type in our cohort due to the sampling limitations, e.g., evaluating individuals on only one type of each surface, which could confound the surface effect with the inherent variability of individual horses.

Beyond the temporal dissociation, several morphometric predictors demonstrated significant correlations with specific gait support stances. A weak negative correlation was observed between pelvic length (tuber coxae to tuber ischii) and the suspension variable. From a biomechanical perspective, a longer pelvic length, characterized by a greater distance between the tuber coxae and the tuber ischii, may be associated with a longer stride and lower limb elevation, as observed in working or endurance horses, in contrast to the patterns seen in horses with a high-stepping trot, where suspension phases are equivalent to diagonal support phases [43,44,45]. Accounting for the historical development of the Criollo horse, it is possible that the selective pressure favored gaits with reduced vertical amplitude, shorter suspension times and triple and monopedal contacts. In contrast, a significant positive correlation was observed between a longer thoracic pastern and an increase in suspension time (*p* = 0.0073), possibly due to a biomechanical “spring-like” function of the distal limb. The pastern acts as a critical system for both shock absorption and propulsion; longer pasterns provide a greater lever arm that facilitates increased elastic deformation of the suspensory apparatus during the loading phase [42,46,47]. Pastern conformation enhances the efficiency of the limb elastic recoil, potentially increasing the suspension phase [48]. Our findings suggest that Criollo horses with longer thoracic pasterns possess a more efficient mechanical advantage for generating the vertical lift required for suspension. Still, these findings need to be evaluated in the context of a larger equine population as well as compared to other breeds.

Pelvic monopedal support in this cohort was weakly influenced by pelvic cannon bone length, humeral (arm) length, and the forearm length (radius and ulna). These findings suggest that both forelimb and hindlimb proportions may play a role in the stability of pelvic monopedal stances during locomotion. Furthermore, pelvic triple support was significantly influenced by the chest width and leg length. Interestingly, thoracic triple support appeared to be exclusively associated with thoracic measurements; both humeral length and the thoracic cannon bone were significantly yet weakly correlated. This compartmentalization of influence suggests that while pelvic support phases may be affected by global body measurements, thoracic support phases are more strictly governed by the anatomy of the forelimb itself. The findings of the present study provide evidence of a functional association regarding the morphometric influence on dynamic gait stability. We observed that thoracic triple support was associated with variables intrinsic to the forelimb, specifically humeral length (*p* = 0.0443) and the third metacarpal bone (*p* = 0.0177). This correlation suggests a degree of segmental autonomy, wherein the stability of the forehand in the Criollo horse is more related to the proportions of the limb itself. In contrast, pelvic support variables exhibited correlations with whole body dimensions, such as chest width (*p* = 0.0143) and leg length (*p* = 0.0134). Such divergence indicates that while thoracic support is more related to the proximal and distal segmental measures of the forelimb, posterior stability and therefore the propulsive unit or ‘engine’ of equine locomotion is contingent upon both trunk conformation and the torque-generating capacity of the pelvic levers. These findings corroborate the kinematic differentiation between the fore and hindlimbs [47], which postulates distinct mechanical roles for each limb pair in managing vertical and propulsive forces during the trot. It is important to note that despite the significative associations, these had either weak (R^2^ ≤ 0.250) or weak-to-moderate (R^2^ ≤ 0.40) correlations, thus further evidence in a larger population is necessary to establish a stronger correlation and causation.

A collected trot should exhibit minimal percentages of tripedal supports (<5% of stride cycle) and diagonal dissociation (<2% of stride cycle) with a small percentage of monopedal stances (~10–15%) [36]. We observed tripedal supports in our group of horses due to brief diagonal overlaps during what likely should have been a bipedal-to-suspension transition. While previously described gaits lacking a suspension phase, with diagonal bipedal support accounting for approximately 60% of the stance duration, are noted in the literature—e.g., the fox-trot [34,49]—the Criollo horse locomotion has a suspension phase, distinguishing it from a fox-trot. Furthermore, the Criollo cohort demonstrated over 60% of diagonal supports, while the fox-trot is characterized by a perceptible amount of lateral bipedal support (roughly 20%), which was negligible or absent in the Criollo horses evaluated. The gait described in this study may represent a distinct Criollo breed locomotion pattern. Despite the limited sample size, our results suggest that the gait of the Criollo horse may not align with the characteristic trot reported in other breeds. The symmetrical sequence typical of the standard trot consisting of diagonal support, suspension, opposite diagonal support, and suspension, where suspension times are comparable to diagonal support times [36], was mostly not represented. Furthermore, unlike the standard trot which excludes non-diagonal phases, our cohort demonstrated monopedal, triple, quadruple, and ipsilateral bipedal contacts.

While equine kinematic studies attempt to minimize environmental interference factors by using consistent riders, equipment, and uniform ground, our study was conducted under conditions reflecting the horse’s normal working environments, which introduces limitations. While the statistical model included rider ID as a random effect to account for variability, the use of a non-standardized ground remains a factor that could influence the results. While we acknowledge these limitations, our results provide valuable insights into the Criollo breed kinematics, contributing both to a better understanding of the breed’s gait under working conditions and offering a baseline for future research involving controlled environments.

## 5. Conclusions

Our findings reveal that the Brazilian Criollo horse locomotor strategy at the “intermediate speed” differs from the symmetrical trot patterns traditionally described in equine biomechanics, being characterized by a distinct diagonal dissociation that is actively modulated by extrinsic and intrinsic factors. We also demonstrate that rather than a fixed gait pattern, the alternation between hindlimb-first and forelimb-first contact could also represent a functional adaptation to ground hardness and head-neck positions (HNP). The magnitude of these shifts, particularly the significant influence of surface compliance and postural changes, reveals a specialized biomechanical efficiency and adaptability related to this breed. Furthermore, the correlation between specific morphometric dimensions and support phase duration suggests that individual conformation plays a role in energy conservation. These results provide a new quantitative baseline for evaluating equine performance and welfare, demonstrating that Criollo horses possibly optimize limb synchrony to mitigate impact and enhance locomotor stability under varying environmental conditions.

## Data Availability

Supplementary data associated with this article can be found, in the online version, at “OSF” in https://osf.io/hs3yt/overview (accessed on 24 March 2026). https://doi.org/10.17605/OSF.IO/HS3YT.

## Supplementary Materials

The following supporting information can be downloaded at: https://www.mdpi.com/article/10.3390/ani16071043/s1.

## Abbreviations

The following abbreviations are used in this manuscript:

HNP	Head and Neck Position
GRF	Ground Reaction Forces
PCA	Principal Component Analysis
ML	Maximum Likelihood
